# Genetic Risk for Attention-Deficit/Hyperactivity Disorder Contributes to Neurodevelopmental Traits in the General Population

**DOI:** 10.1016/j.biopsych.2014.02.013

**Published:** 2014-10-15

**Authors:** Joanna Martin, Marian L. Hamshere, Evangelia Stergiakouli, Michael C. O’Donovan, Anita Thapar

**Affiliations:** a MRC Centre for Neuropsychiatric Genetics and Genomics, Institute of Psychological Medicine and Clinical Neurosciences, Cardiff University School of Medicine, Cardiff; b MRC Integrative Epidemiology Unit, University of Bristol, Bristol, United Kingdom

**Keywords:** Attention-deficit/hyperactivity disorder, autism spectrum disorder, Avon Longitudinal Study of Parents and Children (ALSPAC), genetics, pragmatic language, social communication

## Abstract

**Background:**

Attention-deficit/hyperactivity disorder (ADHD) can be viewed as the extreme end of traits in the general population. Epidemiological and twin studies suggest that ADHD frequently co-occurs with and shares genetic susceptibility with autism spectrum disorder (ASD) and ASD-related traits. The aims of this study were to determine whether a composite of common molecular genetic variants, previously found to be associated with clinically diagnosed ADHD, predicts ADHD and ASD-related traits in the general population.

**Methods:**

Polygenic risk scores were calculated in the Avon Longitudinal Study of Parents and Children (ALSPAC) population sample (*N* = 8229) based on a discovery case-control genome-wide association study of childhood ADHD. Regression analyses were used to assess whether polygenic scores predicted ADHD traits and ASD-related measures (pragmatic language abilities and social cognition) in the ALSPAC sample. Polygenic scores were also compared in boys and girls endorsing any (rating ≥1) ADHD item (*n* = 3623).

**Results:**

Polygenic risk for ADHD showed a positive association with ADHD traits (hyperactive-impulsive, *p* = .0039; inattentive, *p* = .037). Polygenic risk for ADHD was also negatively associated with pragmatic language abilities (*p* = .037) but not with social cognition (*p* = .43). In children with a rating ≥1 for ADHD traits, girls had a higher polygenic score than boys (*p* = .003).

**Conclusions:**

These findings provide molecular genetic evidence that risk alleles for the categorical disorder of ADHD influence hyperactive-impulsive and attentional traits in the general population. The results further suggest that common genetic variation that contributes to ADHD diagnosis may also influence ASD-related traits, which at their extreme are a characteristic feature of ASD.

Attention-deficit/hyperactivity disorder (ADHD) is a highly heritable neurodevelopmental disorder characterized by early-onset, developmentally inappropriate inattentive, hyperactive, and impulsive behaviors (1). The disorder occurs more frequently in boys, with a male-to-female ratio of about 3–7:1 2, 3. Similar to other common disorders, the genetic architecture of ADHD is complex, with rare and common variants involved (4). Although clinical diagnoses are defined categorically, ADHD psychopathology can also be viewed dimensionally, with inattentive and hyperactive-impulsive symptoms distributed continuously in the general population (5). Twin and epidemiological studies have shown that heritability estimates for dimensional ADHD are similar across a variety of cutoff points 6, 7. This similarity in heritability estimates indicates that genetic factors act throughout the full distribution of ADHD symptoms. However, the postulated relationship between dimensional measures of ADHD in the population and clinical diagnoses has not yet been confirmed at the level of molecular genetics.

It has become clear in more recent years that the boundaries between different neurodevelopmental and psychiatric disorders are not clear-cut, as exemplified by the observed clinical and genetic overlap between ADHD and other disorders. Rates of co-occurrence are especially high for ADHD and autism spectrum disorder (ASD), another highly heritable neurodevelopmental disorder, characterized by social communication and interaction deficits as well as restrictive and repetitive behaviors (8). Studies of children with clinical diagnoses have found that large (>500 kb), rare (<1% frequency) copy number variants in ADHD show significant overlap with copy number variant loci previously implicated in ASD 9, 10, although a more recent collaborative cross-phenotype analysis found no clear common genetic overlap in diagnosed ADHD and ASD cases (11). Similar to ADHD, ASD can also be viewed dimensionally (12), and twin studies have found that ADHD and ASD traits share common genetic influences in the general population as well as at the quantitative extreme 13, 14, 15, 16, 17, 18, 19. These studies suggest that genetic variants associated with the diagnosis of ADHD might also contribute to population variation in ASD-related trait measures.

Previous research has suggested that children with a clinical diagnosis of ADHD (*n* = 452) differ from control subjects (*n* = 5081) on the basis of a polygenic risk score, an aggregate score of thousands of common alleles of very small effect that together form an index of genetic risk for ADHD (20). In the present study, we tested the hypothesis that en masse common genetic variants that confer risk for a clinical diagnosis of ADHD are associated with ADHD traits in the general population. Given the established clinical and genetic overlap between ADHD and ASD 13, 14, 16, we also analyzed the secondary hypothesis that en masse ADHD common genetic variants are also associated with ASD-related social communication traits in the general population.

## Methods and Materials

### Target Population Sample

The Avon Longitudinal Study of Parents and Children (ALSPAC) is a large, well-characterized longitudinal data set 21, 22. ALSPAC originally recruited pregnant women (*N* = 14,541) residing in Avon, England, with expected delivery dates of April 1, 1991–December 31, 1992. An additional 713 eligible children whose mothers did not enroll during pregnancy were enrolled after age 7, resulting in a total sample of 14,701 of children alive at age 1 year. Full data (phenotypic and genotypic) were available for up to 5661 children, depending on the outcome variables. Children with >30% missing items on any outcome variable were excluded from analyses of that variable. The study website (http://www.bris.ac.uk/alspac/researchers/data-access/data-dictionary/) contains details of all available data. Ethical approval for the study was obtained from the ALSPAC Ethics and Law Committee and local research ethics committees.

#### Phenotypic Measures

Data on ADHD traits were collected when participants were ~7 years, 7 months old, using the parent Development and Well-Being Assessment (DAWBA) (23). For each ADHD item, parents marked boxes to say whether their child showed the behavior; these were coded as follows: 0 for “no,” 1 for “a little more than others,” and 2 for “a lot more than others.” A total ADHD trait score was calculated by summing these responses to give a possible range of 0–36. Scores were also calculated for inattentive and hyperactive-impulsive ADHD traits separately (with a possible range of 0–18 each).

Social communication traits were assessed using the Social and Communication Disorders Checklist (SCDC) (24) and the pragmatic language scales of the Children’s Communication Checklist (CCC) (25). A quantitative measure of restricted, repetitive behaviors was not available. Both the CCC and the SCDC have been shown to have good predictive reliability for a clinical diagnosis of ASD in the ALSPAC sample (26). The CCC shows good interrater reliability (.80), internal consistency (.80–.87), and validity for language problems (25), and the SCDC shows good internal consistency (.93), high test-retest reliability (.81), and validity for a diagnosis of ASD (24). The SCDC assesses social cognition and understanding, whereas the CCC pragmatic language scales measure ability to use language in a social context. Previous research has shown that children with ADHD or ASD have lower pragmatic language ability scores than control subjects with typical development, but children with ASD have lower scores than children with ADHD (27).

The SCDC was assessed at the same time as the DAWBA ADHD measures. Parents were asked to judge how much 12 descriptions applied to their child’s behavior. The responses were coded as follows: 0 for “not true,” 1 for “quite/sometimes true,” and 2 for “very/often true.” A total SCDC score was calculated by summing these responses (with a possible range of 0–24).

An abridged version of the CCC was used to assess language abilities at ~9 years, 7 months of age. Parents were asked to rate whether statements about their child were “certainly true,” “somewhat true,” or “not true,” which were coded as 0, 1, and 2. The following subscales were summed to generate a pragmatic language abilities score: inappropriate initiation, coherence, stereotyped conversation, conversational context, and conversational rapport. Subscale scores were based on six to eight items each. The pragmatic language total score was obtained for children with data available for each subscale. Because the CCC measures language abilities, lower scores suggest pragmatic language deficits.

Information on DSM-IV ADHD diagnoses is available based on the DAWBA at ~7 years of age. Data on ASD diagnoses are available based on clinical records, using a clinician’s diagnosis of ASD (28). Prorated scores were used for measures with <30% missing items.

#### Genetic Data

After quality control (QC), genome-wide data for 500,527 single nucleotide polymorphisms (SNPs) were available for 8229 of the children, of whom 4213 (51.2%) were boys. Details of QC procedures are provided in Supplement 1.

### Discovery Clinical Sample for Generating ADHD Polygenic Risk Scores

The analytic method described by the International Schizophrenia Consortium (29) was used to identify ADHD risk alleles in a discovery genome-wide association study (GWAS) from which polygenic risk scores were derived in the ALSPAC subjects. A published GWAS of British and Irish children with a confirmed DSM-IV research diagnosis of ADHD (*n* = 727) and population control subjects (*n* = 5081) was used to define risk alleles. This clinical sample was selected as the primary discovery sample because it is similar to the ALSPAC general population in ethnicity and underwent similar diagnostic assessment procedures. The ascertainment of DNA samples, QC procedures, and GWAS results were described in detail previously (4). This GWAS was based on 502,702 SNPs after strict QC. Following the International Schizophrenia Consortium study, alleles that were more common in cases than controls at SNPs showing evidence for association at the very relaxed threshold *p* < .5 were considered risk alleles.

### Generating Polygenic Scores

Full details are available in Supplement 1. In brief, SNPs in approximate linkage equilibrium in the ALSPAC genome-wide data were identified using the PLINK software, available for free download at http://pngu.mgh.harvard.edu/~purcell/plink/ (30). From this set of SNPs, we retained alleles that showed evidence for weak association (*p* < .5) in the discovery ADHD GWAS and used those to calculate a polygenic score for each individual in ALSPAC using PLINK (30). The polygenic scores were standardized using *z* score transformations.

### Data Analysis Strategy

In the ALSPAC sample, children with ADHD or ASD diagnoses were compared with each other and with the remainder of the sample on ADHD, SCDC, and CCC traits, using Student *t* test. Girls and boys were also compared. Analyses were conducted on the 8229 ALSPAC children with full genetic data available after all QC.

**Figure 1:**
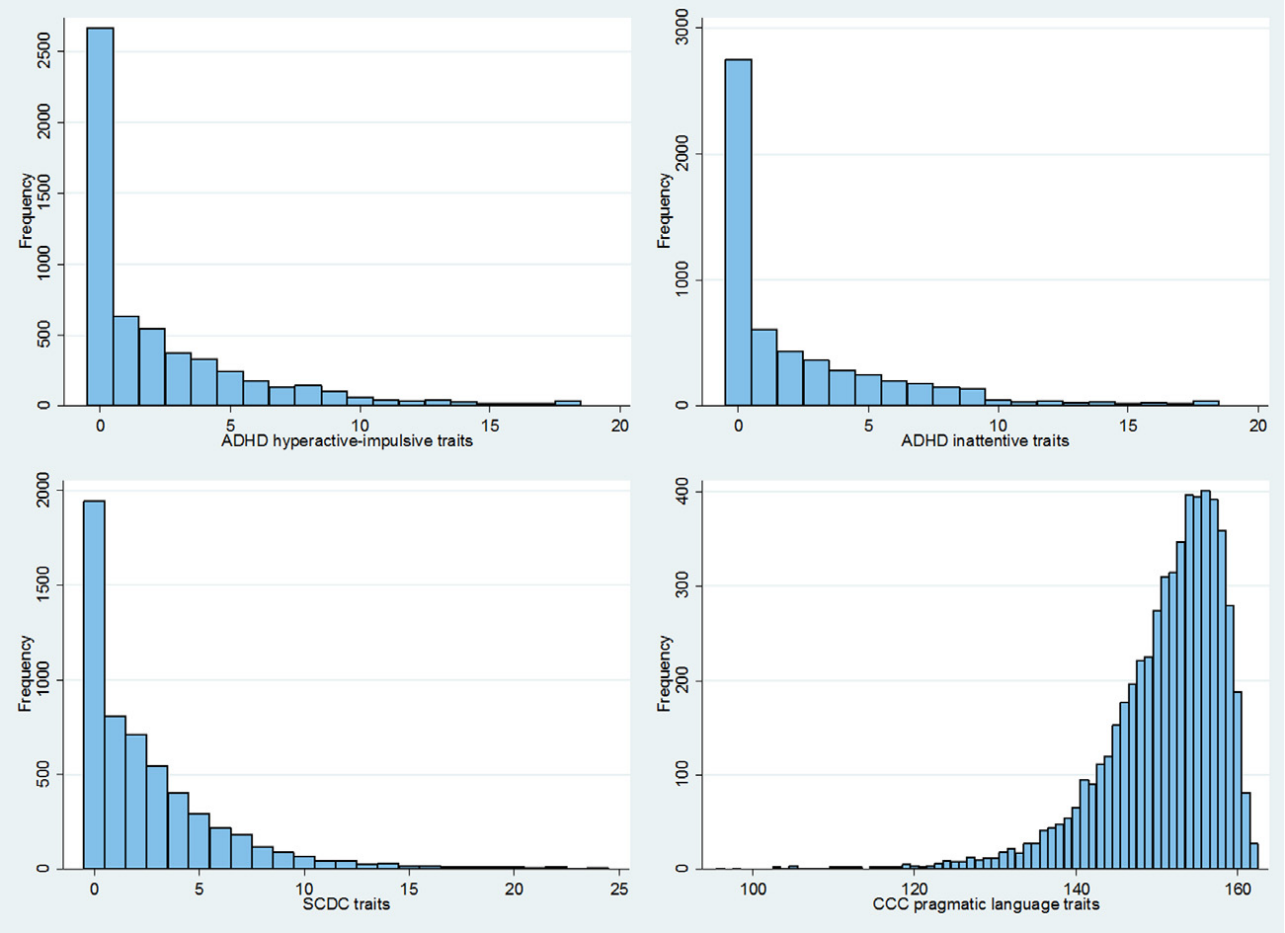
Histograms of attention-deficit/hyperactivity disorder and social communication traits. ADHD, attention-deficit/hyperactivity disorder; CCC, Children’s Communication Checklist; SCDC, Social and Communication Disorders Checklist.

As a result of a strongly negatively skewed distribution of the CCC pragmatic language data, variables were transformed (ln x + 1) and linear regression analyses were performed to test for association with ADHD polygenic score. The ADHD and SCDC traits were highly positively skewed, contained an excess of zero values, and could not be transformed to normality (see Figure 1 for variable distributions). Analyzing such data using standard linear regressions may yield biased estimates of parameters and increased type I and II error rates 31, 32. The distribution of data was better explained by a negative binomial than a Poisson distribution of simulated data with the same mean and number (Figure S1 in Supplement 1). These data were analyzed using zero-inflated negative binomial (ZINB) regression models. Gender was included as a covariate in all models.

The ZINB model consists of two submodels that allow for a distribution with an inflated number of individuals with values of zero: 1) logistic regression model of an unobserved dichotomous outcome to predict who has a score = 0 and who has a score >0 and 2) negative binomial model of the continuous outcome in individuals having a score ≥0. Likelihood ratio tests were used to determine an overall *p* value for each ZINB model compared with a null model, which included gender but not polygenic score. The ZINB analyses were performed using Mplus version 7 (Muthén & Muthén, Los Angeles, California) (33).

For each association test, the amount of variance explained was calculated as the difference of Nagelkerke pseudo-*R*^*2*^ in the full model compared with the null model. Given the non-independence of the outcome variables, all results are interpreted using a significance threshold of *p* < .05. Given that previous analysis of polygenic scores for ADHD in a clinical sample of children with ADHD showed that girls had higher polygenic scores than boys (20), a Student *t* test was used to test whether polygenic scores in children rating positive for any (rating ≥1) ADHD trait in the target sample were significantly higher in girls than in boys.

Where significant associations were observed, secondary analyses were run to determine whether the same associations could be detected for traits at a later time point (~10 years, 8 months years of age). Replication was sought using a second ADHD GWAS discovery sample—the Psychiatric Genomics Consortium (34). This sample contained 2064 trios, 896 cases, and 2455 control individuals from four individual studies. There were 54 cases (2% of the cases in this second sample) that overlapped with the main discovery sample, but they could not be removed because only the summary statistics were available for this analysis.

## Results

### Sample Phenotypic Characteristics

**Figure 2:**
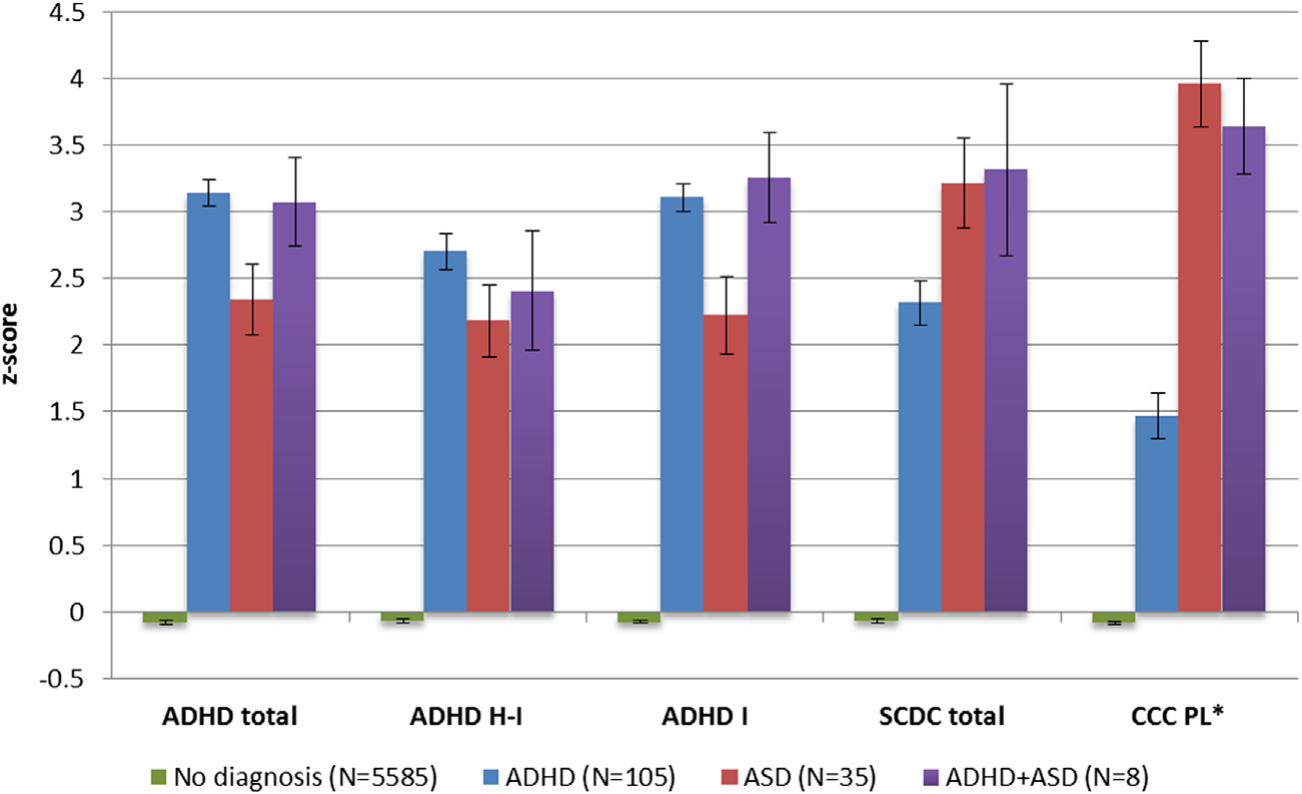
Mean *z* scores of ADHD and social communication outcomes, displayed by diagnostic group. Error bars represent SEM. ⁎Scores reversed. ADHD, attention-deficit/hyperactivity disorder; ASD, autism spectrum disorder; CCC PL, Children’s Communication Checklist pragmatic language; H-I, hyperactive-impulsive; I, inattentive; SCDC, Social and Communication Disorders Checklist.

Figure 2 presents descriptive statistics of the trait measures in children with no ADHD or ASD (*n* = 5585), children with a diagnosis of ADHD (*n* = 105), children with a diagnosis of ASD (*n* = 35) or children with both ADHD and ASD (*n* = 8). Of the children with a diagnosis of ADHD, 7.1% also had a diagnosis of ASD; of the children with ASD, 36.4% also had ADHD. This overlap was greater than would be expected by chance (χ^2^ = 136.0, *p* < .001).

As expected, ADHD traits were higher in children with a diagnosis of ASD than in children without ADHD or ASD (hyperactive-impulsive, *t* = 13.03, *p* < .001; inattentive, *t* = 13.12, *p* < .001). Children with ASD had lower levels of inattentive traits than children with ADHD (*t* = −3.50, *p* < .001) but did not differ significantly in terms of hyperactive-impulsive traits (*t* = −1.70, *p* = .09).

Children with a diagnosis of ADHD had significantly higher SCDC scores (*t* = 26.71, *p* < .001) and lower CCC pragmatic language scores (*t* = −11.45, *p* < .001) than children without ADHD or ASD but had lower SCDC scores (*t* = −2.45, *p* = .016) and higher pragmatic language ability scores (*t* = 6.17, *p* < .001) than children with ASD. The ADHD and social communication outcomes were moderately correlated (Table 1). Compared with boys, girls had significantly lower scores for ADHD (hyperactive-impulsive, *t* = −12.48, *p* < .001; inattentive, *t* = −13.06, *p* < .001) and SCDC (*t* = −9.50, *p* < .001) and higher CCC pragmatic language ability scores (*t* = 6.44, *p* < .001).

**Table 1:** Pearson Correlation Coefficients of ADHD and Social Communication Outcome Measures

	ADHD HI	ADHD I	ADHD Total	SCDC
ADHD I	.71			
ADHD Total	.92	.93		
SCDC	.65	.58	.66	
CCC PL	−.51	−.48	−.53	−.51

All associations significant at *p* < .001.

ADHD, attention-deficit/hyperactivity disorder; CCC PL, Children’s Communication Checklist pragmatic language; H-I, hyperactive-impulsive; I, inattentive; SCDC, Social and Communication Disorders Checklist.

### Polygenic Score Analysis of ADHD and ASD-Related Social Communication Traits

The ADHD polygenic scores were based on 49,595 SNPs and were normally distributed in the ALSPAC sample (*N* = 8229). Among children with any ADHD traits (rating ≥1; *n* = 3623), girls had a higher polygenic score than boys (*t* = 2.94, *p* = .003, Cohen’s *d* = .098). This finding is not attributable to an overall population difference on polygenic score by gender (*t* = 1.59, *p* = .11; *N* = 8229). Gender was included as a covariate in all further analyses.

Results of associations of ADHD polygenic score with the ADHD and social communication outcomes are shown in Table 2. The ZINB models show that ADHD polygenic risk predicted ADHD total scores (*R*^*2*^ = .005, *p* = .0026), hyperactive-impulsive traits (*R*^*2*^ = .002, *p* = .0039), and inattentive traits (*R*^*2*^ = .002, *p* = .037). The ZINB models indicate that the association signal comes from the zero-inflated part (part 1) of the model for all ADHD outcomes.

**Table 2:** Associations of Polygenic Score with ADHD and ASD-Related Phenotypes in ALSPAC

Outcome	*n*	ZINB Count Outcome			ZINB Zero-Inflated Outcome			ZINB Overall *p*	ZINB Overall *R*^2^	Linear Regression^a^			
		β	SE	*p*	β	SE	*p*			β	SE	*p*	*R*^2^
ADHD Total Traits	5661	.11	.10	.30	−.06	.02	.005	.0026^b^	.005^b^	.032	.013	.013	.001
ADHD Hyperactive-Impulsive Traits	5661	.15	.13	.24	−.05	.02	.024	.0039^b^	.002^b^	.037	.013	.005	.001
ADHD Inattentive Traits	5656	.05	.13	.71	−.05	.02	.019	.037^b^	.002^b^	.023	.013	.084	.001
SCDC Total Score	5653	.15	.19	.45	.02	.04	.67	.43^b^	<.001^b^	.012	.013	.35	.0002
CCC Pragmatic Language Score	5641									−.028	.013	.037^b^	.001^b^

All analyses used gender as a covariate. Polygenic scores derived using a threshold of *p* < .5 in the discovery sample genome-wide association study results (see text).

ADHD, attention-deficit/hyperactivity disorder; ALSPAC, Avon Longitudinal Study of Parents and Children; CCC, Children’s Communication Checklist; SCDC, Social and Communication Disorders Checklist; ZINB: zero-inflated negative binomial.

a Linear regression results of ADHD and SCDC phenotypes included only for ease of interpretation.

b Main result.

To explore further the contribution of polygenic scores to ADHD trait levels in subjects with nonzero scores, the population was split into three arbitrary groups, based on increasing trait score: children who scored 0 (*n* = 2038), children with low levels of ADHD (score = 1–11; *n* = 2817), and children with moderate-to-high levels of ADHD (score ≥12; *n* = 806). Analysis of variance showed a significant group difference (*F* = 4.66, *p* = .010), and post hoc tests revealed that children with no ADHD traits had a lower mean polygenic score than children with ADHD scores of 1–11 (*p* = .022) and children with scores ≥12 (*p* = .037). The difference between the two other groups was not significant (*p* = .80).

The ADHD polygenic scores showed a significant association with lower CCC pragmatic language scores (β = −.028, *p* = .037). Exploration of whether findings were attributable to specific CCC subscales showed association with lower scores on the “inappropriate initiation” and “conversational context” subscales (β = −.034, *p* = .009, and β = −.034, *p* = .010, respectively) but not with “coherence,” “stereotyped conversation,” and “conversational rapport” (all *p* > .05). No association was found between polygenic score and SCDC total score (*p* > .05).

Structural equation modeling with ADHD and pragmatic language as correlated outcomes confirmed that both constructs are independently predicted by polygenic score (Figure S2 in Supplement 1). The amount of variance explained (*R*^*2*^) for all models was very small, although this estimate does not reflect the true magnitude of the genetic overlap because it is highly sensitive to sample size (29). Including the 10 EIGENSTRAT principal components as covariates in the analyses did not affect the results (Table S2 in Supplement 1).

### Testing Associations at Age 10

The observed association between polygenic score and ADHD (at ~7.5 years of age) could also be seen at the later time point (~10.5 years of age, *n* ≥ 5495) for total ADHD traits (*R*^*2*^ = .004, *p* = .012) and hyperactive-impulsive traits (*R*^*2*^ = .003, *p* = .039), with weak association with inattentive traits (*R*^*2*^ = .002, *p* = .055) (Table 3). Among children with any ADHD traits at age 10 (≥1; *n* = 3316), girls had a higher polygenic score than boys (*t* = 2.35, *p* = .019, Cohen’s *d* = .082).

**Table 3:** Secondary Analysis—Associations of Polygenic Score with ADHD at Age 10 Years

Outcome	*n*	ZINB Count Outcome			ZINB Zero-Inflated Outcome			ZINB Overall *p*	ZINB Overall *R*^2^	Linear Regression^a^			
		β	SE	*p*	β	SE	*p*			β	SE	*p*	*R*^*2*^
ADHD Total Traits	5500	−.05	.12	.68	−.06	.02	.003	.012^b^	.004^b^	.087	.086	.31	.0002
ADHD Hyperactive-Impulsive Traits	5505	−.15	.25	.53	−.06	.02	.012	.039^b^	.003^b^	.019	.043	.66	3.4E-05
ADHD Inattentive Traits	5495	.02	.14	.90	−.04	.02	.021	.055^b^	.002^b^	.076	.051	.14	.0004

All analyses used gender as a covariate. Polygenic scores derived using a threshold of *p* < .5 in the discovery sample genome-wide association study results (see text).

ADHD, attention-deficit/hyperactivity disorder; ASD, autism spectrum disorder; ZINB, zero-inflated negative binomial.

a Linear regression results included only for ease of interpretation.

b Main result.

### Replication Using Second Discovery Sample

Polygenic scores based on the second discovery sample (34) were not significantly associated with ADHD traits at age 7 (*p* > .05) but did show an association at age 10 with total ADHD traits (*R*^*2*^ = .001, *p* = .019) and hyperactive-impulsive traits (*R*^*2*^ < .001, *p* = .018), with weak association with inattentive traits (*R*^*2*^ < .001, *p* = .055) (Table 4). Polygenic scores based on the second discovery sample also showed an association with the CCC “conversational context” subscale (β = −.031, *p* = .017) but showed no association with the CCC “inappropriate initiation” subscale (β = −.006, *p* = .37).

**Table 4:** Replication Analyses—Associations of Polygenic Score Based on Second Discovery Sample with ADHD at Both Time Points

Time	Outcome	*n*	ZINB Count Outcome			ZINB Zero-Inflated Outcome			ZINB Overall *p*	ZINB Overall *R*^*2*^	Linear Regression^a^			
			β	SE	*p*	β	SE	*p*			β	SE	*p*	*R*^*2*^
Age 7	ADHD Total Traits	5661	.11	.11	.30	−.02	.02	.338	.20^b^	.001^b^	.023	.13	.052	.001
	ADHD Hyperactive-Impulsive Traits	5661	.05	.10	.58	−.03	.02	.20	.26^b^	<.001^b^	.020	.013	.12	.0004
	ADHD Inattentive Traits	5656	.18	.20	.39	−.02	.02	.44	.17^b^	<.001^b^	.027	.013	.043	.001
Age 10	ADHD Total Traits	5500	.27	.24	.26	−.02	.02	.45	.019^b^	<.001^b^	.26	.087	.003	.002
	ADHD Hyperactive-Impulsive Traits	5505	.30	.39	.44	−.01	.02	.56	.018^b^	<.001^b^	.13	.043	.003	.002
	ADHD Inattentive Traits	5495	.29	.33	.38	−.01	.02	.65	.055^b^	<.001^b^	.13	.052	.015	.001

All analyses used gender as a covariate. Polygenic scores derived using a threshold of *p* < .5 in the discovery sample genome-wide association study results (see text).

ADHD, attention-deficit/hyperactivity disorder; ZINB, zero-inflated negative binomial.

a Linear regression results included only for ease of interpretation.

b Main result.

In children with ADHD trait scores ≥1 at age 7, there was a trend for girls to have a higher polygenic score than boys, calculated using this second discovery sample (*t* = 1.80, *p* = .071, Cohen’s *d* = .060). At age 10, girls had significantly higher polygenic scores than boys (*t* = 2.18, *p* = .029, Cohen’s *d* = .076).

## Discussion

As hypothesized, this study found that ADHD polygenic score, based on common genetic variants previously found to be associated with risk of a clinical diagnosis of ADHD, was also associated with ADHD traits measured at ages 7 and 10 years in the general population. This finding is important because it provides support at the level of molecular genetics for the hypothesis that ADHD represents the extreme end of traits present in the general population 6, 7. The results also support the relevance of common genetic variants to ADHD (4), extending findings by showing they also act on nonclinical ADHD traits in a community sample.

The exploratory analysis of variance results show that polygenic score, which is derived from common genetic variants relevant to clinical (i.e., severe) ADHD, predicted both low levels and high levels of ADHD traits in the general population. The ZINB analysis suggested that the association signal between polygenic score and ADHD traits originates from the zero-inflated part of the model (i.e., whether ADHD trait score was zero or nonzero). This result might be due to greater power at the lower end of ADHD traits, as progressively fewer children have higher levels of ADHD traits.

Consistent with previous literature in clinical and general population samples 15, 16, 35, children with diagnoses of ADHD had more ASD-related social communication problems than children without a diagnosis of ADHD or ASD, whereas children with ASD had more ADHD traits than children without either diagnosis. Although children with ADHD had higher inattentive traits than children with ASD, levels of hyperactive-impulsive traits in these two groups did not differ significantly. However, this finding could have been due to low power because few children in the ALSPAC cohort had a clinical ASD diagnosis.

Results of the genetic analysis also suggest that risk alleles for ADHD may contribute to phenotypic traits in the general population, beyond core ADHD features. Polygenic risk scores previously found to be associated with diagnosis of ADHD were also nominally associated with pragmatic language abilities in the general population but not with social cognition traits, as indexed by SCDC scores.

Secondary exploratory analyses suggested that the association of ADHD polygenic risk with pragmatic language score was driven by scores on the “inappropriate initiation” and “conversational context” subscales of the CCC. Some items in the “inappropriate initiation” subscale may tap into impulsive ADHD behaviors (in particular, the CCC item “he/she talks too much”), but items in the “conversational context” subscale (e.g., “he/she can understand sarcasm” or “he/she says things which are tactless or socially inappropriate”) have no apparent link with ADHD features. Overall, the findings suggest that risk variants for ADHD may have pleiotropic effects on closely related but conceptually different neurodevelopmental traits in the general population. These findings also support findings from a twin study, in which ADHD traits at age 8 shared genetic effects and were most associated with ASD communication difficulties, rather than ASD social difficulties or stereotyped behaviors (17).

One possible advantage of the primary discovery ADHD sample used to derive risk alleles, over the replication sample, is its similarity to the ALSPAC cohort in terms of ancestry and geography, but nevertheless the sample was relatively small (4). Analyses using a second, larger ADHD sample (34) showed a partial replication of the primary analysis. Polygenic scores based on this sample predicted ADHD traits at age 10, although not at age 7. Similarly, although polygenic scores derived from the second ADHD dataset predicted pragmatic language problems, as assessed using the CCC “conversational context” subscale, they did not predict variation on the CCC “inappropriate initiation” subscale. These replication results suggest that the associations of ADHD polygenic score with ADHD traits and pragmatic language problems are robust. However, further replication is necessary to rule out possible type I error conclusively. These results also further highlight the fact that absence of clear individually associated loci in current GWAS of ADHD reflects inadequate power of the GWAS samples, rather than an absence of common susceptibility variants.

Although we found an association between ADHD polygenic score and pragmatic language abilities, there was no association with social cognition, as measured by the SCDC. A more recent collaborative cross-phenotype analysis suggested that common GWAS variants do not contribute to the overlap in diagnoses of ADHD and ASD (11). Nevertheless, evidence in twin studies is consistent in finding high heritability for neurodevelopmental trait measures and in showing shared genetic influences on ADHD and ASD 6, 7, 16. It is too early to discount the contribution of common variants to the overlap of ADHD and ASD, particularly in terms of continuously distributed traits. The current study points to a possible overlap between susceptibility to clinically diagnosed ADHD and pragmatic language difficulties at a trait level in the general population.

As expected, boys in the ALSPAC cohort had higher ADHD trait scores than girls 16, 36, 37. However, a novel observation was that girls had higher polygenic scores than boys in the group of children with any ADHD symptoms at either age. For polygenic scores based on the second discovery sample, there was a trend toward similarly higher scores in girls at age 7 and significantly higher scores at age 10 years. These results support the previous observation that in children with a diagnosis of ADHD, girls have higher polygenic scores than boys (20). One limitation of the earlier study is that it was based on a clinical sample, so the gender difference may have reflected referral bias (i.e., referred girls on average may have had a more severe phenotype). The present finding in an epidemiological sample argues against that bias and suggests a different liability threshold for girls than boys, with girls requiring a more extreme load of risk factors to manifest ADHD. This suggestion is consistent with non–molecular based studies; for example, one study observed that siblings of girls with ADHD have more ADHD symptoms than siblings of boys with ADHD (38). Similar findings have been reported in nonidentical twin children with ASD (39).

A limitation of this study was that although the SCDC and CCC measures of social cognition and pragmatic language are predictive of a clinical diagnosis of ASD in the sample (26), they are not strictly measures of the specific deficits required for an ASD diagnosis. Also, no reliable quantitative measure of restrictive and repetitive behaviors was available. The finding of an association between ADHD polygenic score and pragmatic language deficits is potentially also relevant to the new DSM-5 category of “social communication disorder” (40).

Because the ALSPAC cohort is longitudinal, the sample is affected by attrition. Previous studies have determined that predictors of attrition include socioeconomic and pregnancy factors as well as presence of behavioral difficulties, including ADHD, in the study child (41). Assuming that attrition results from the behavioral manifestation of genetic risk, resultant attrition bias is likely to reduce the correlation between risk scores and traits. Multiple imputation methods have been used previously for missing ALSPAC data but do not appear to alter association patterns (42).

As a result of the relatively small ADHD GWAS discovery sample sizes, power to detect susceptibility variants is low, and aggregate scores based on GWAS are likely to be based on a poor signal-to-noise ratio 4, 34. This is a possible explanation for the relatively small amount of phenotype variance explained by polygenic scores in the current study, estimates of explained variance in this form of analysis being strongly affected by discovery sample size. Another limitation of the current study is that a small number (*n* = 54) of cases overlapped in both discovery samples. Although *p* < .5 is frequently used as a threshold for calculating polygenic scores 29, 43, 44, 45, this is largely a convention established on the basis of the optimal threshold in the study of schizophrenia that inspired the wider application of polygenic score analysis (29). As shown by modeling in that study, the optimal threshold depends on both genetic architecture and sample size, and other thresholds have the potential to show greater effects. A sensitivity analysis in the present study using a variety of *p* value thresholds for calculating polygenic scores demonstrated that observed effects are consistent across various thresholds (Figure S3 in Supplement 1).

In summary polygenic risk previously found to be associated with clinical ADHD diagnosis predicted inattentive and hyperactive-impulsive traits in a general population sample. This study also indicates that common genetic variants associated with ADHD may be associated with pragmatic language ability in the general population, a trait measure that is distinct from the core deficits of ADHD. The approach of testing genetic risks that contribute to dimensions that cut across diagnostic categories, rather than using DSM diagnoses, is in line with the Research Domain Criteria framework (46) and is likely to be a valuable approach for future neurodevelopmental and psychiatric research. As the power of GWAS increases, this method has the potential to explore the biological overlap of these traits further.
