# Supplementary material for: Genetic Risk for Attention-Deficit/Hyperactivity Disorder Contributes to Neurodevelopmental Traits in the General Population
Source: Biol Psychiatry. 2014 Oct 15;76(8):664–71. doi: 10.1016/j.biopsych.2014.02.013 (PMC4183378; doi:10.1016/j.biopsych.2014.02.013)
Supplement: Supplementary file 1 — Supplementary Material [file mmc1.pdf]

Genetic Risk for Attention-Deficit/Hyperactivity Disorder Contributes to Neurodevelopmental Traits in the General Population

Supplementary Materials

Genetic Data - Avon Longitudinal Study of Parents and Children (ALSPAC)

A total of 9,912 ALSPAC children were genotyped using the Illumina HumanHap550 quad genome-wide single nucleotide polymorphism (SNP) genotyping platform by 23andMe subcontracting the Wellcome Trust Sanger Institute, Cambridge, UK and the Laboratory Corporation of America, Burlington, NC, USA. Individuals were excluded from further analysis on the basis of having incorrect gender assignments; minimal or excessive heterozygosity (<0.320 and >0.345 for the Sanger data and <0.310 and >0.330 for the LabCorp data); disproportionate levels of individual missingness (>3%); evidence of cryptic relatedness (>10% identity by descent (IBD)) and being of non-European ancestry (as detected by a multidimensional scaling analysis seeded with HapMap 2 individuals, EIGENSTRAT analysis revealed no obvious population stratification and genome-wide analyses with other phenotypes indicate a low genomic inflation factor, lambda (λ)). SNPs with a minor allele frequency of <1% and call rate of <95% were removed. Furthermore, only SNPs which passed an exact test of Hardy–Weinberg equilibrium ($p > 5\times10^{-7}$) were considered for further use. After quality control (QC), genome-wide data were available for n = 8,231 of the children. The data set contained 500,527 SNPs after QC.

A large proportion of the children in the discovery sample (n = 559; 70%) and all of the target ALSPAC sample were recruited from geographically nearby regions (Wales and Southwest England) and also the individuals overlapped in age. Therefore, an IBD analysis was conducted using PLINK (1) to ensure that there were no related individuals in both samples. Individuals in ALSPAC who showed IBD ≥ 12% in relation to any individual in the discovery sample (n = 2) were removed from analyses, leaving a final sample of n = 8,229 children in ALSPAC.

Calculation of Polygenic Scores

The analysis was confined to autosomal SNPs. SNPs in relative linkage equilibrium in the ALSPAC genome-wide data were selected using a sliding window of 200 SNPs, moving it along the genome 5 SNPs at a time and dropping a SNP when the pair-wise estimate of linkage disequilibrium (R^2) exceeded 0.2, using the command (--indep-pairwise 200 5 0.2) in PLINK (1), giving a list of 101,200 SNPs. Corresponding *p*-values, associated risk alleles and odds ratios were identified for the selected SNPs in the discovery sample, if available. In line with previous studies (2-6), a primary threshold of $p < 0.5$ was used to select alleles more common in cases than controls from the discovery sample. These identified SNPs were used to calculate a polygenic score for each individual in ALSPAC, corresponding to the mean number of score alleles (weighted by odds ratio) across the set of SNPs, using the PLINK command (--score), with imputation of missing genotypes in PLINK (1). Polygenic scores were also calculated at a variety of other *p*-value thresholds to test the sensitivity of observed results. Please see Table S1 below for the number of SNPs at each threshold.

Table S1. Number of SNPs from discovery sample mapped to alleles & used to calculate polygenic scores at each threshold.

p-value threshold	Discovery sample 1 (7)	Discovery sample 2 (8)
$p < 1$	96554	
$p < 0.5$	49595	47226
$p < 0.4$	40060	
$p < 0.3$	30542	
$p < 0.2$	20746	
$p < 0.1$	10687	
$p < 0.05$	5417	
$p < 0.01$	1193	

Other Details

The genotyping arrays for the primary discovery sample (7) were the Illumina Human660W-Quad BeadChip for the cases and the Illumina Human 1.2M BeadChip for controls. The second discovery sample was a meta-analysis of studies conducted on several platforms (Illumina 550K, Perlegen 600K, Affymetrix 500K and Illumina 1M Duo), which were imputed using data from phase 3 of the HapMap project (8).

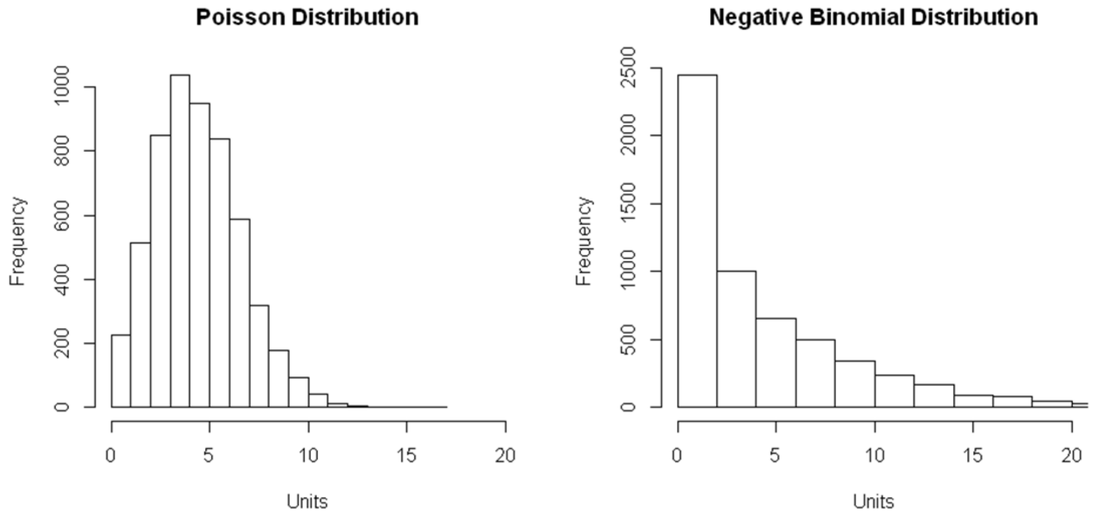

Figure S1. Simulated data showing Poisson and negative binomial distributions. Simulations of an expected typical Poisson distribution (left) and a negative binomial distribution (right) in R, using the sample size (*n* = 5,661) and mean (mean = 4.9) of the total attention-deficit/hyperactivity disorder traits in the ALSPAC sample.

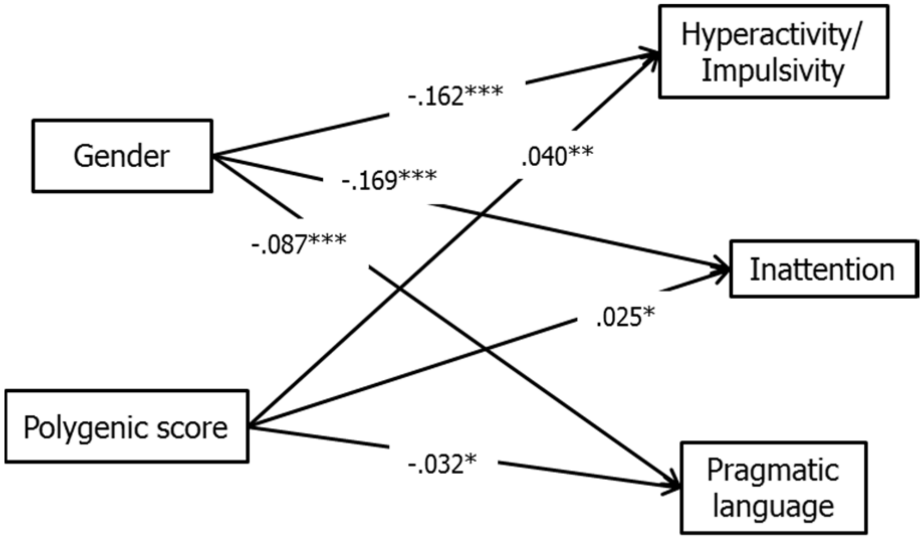

Figure S2. Structural equation modeling of polygenic score predicting multiple correlated outcomes. Structural equation modeling allows for the simultaneous assessment of relationships between multiple predictors and outcomes. This was performed in Mplus version 7. The model estimator used was 'MLR', which provides full information maximum likelihood estimation with robust standard errors, using all available data for the model. Given that the neurodevelopmental outcomes are correlated (coefficients and arrows not shown), modeling the association of polygenic score with all outcomes simultaneously can be used to determine the unique effect of polygenic score on each of these outcomes. Analysis was based on n = 6423. Standardized path coefficients are shown. All paths were estimated and no fit statistics are available due to model saturation. *$p < 0.05$, **$p < 0.01$, ***$p < 0.001$.

Table S2. Associations of polygenic score with ADHD and ASD-related phenotypes in ALSPAC (all analyses using gender and all 10 principal components from EIGENSTRAT analyses as covariates).

Outcome	*n*	ZINB count outcome			ZINB zero-inflated outcome			ZINB overall *p*	ZINB overall R^2	Linear regression*			
		β	SE	*p*	β	SE	*p*			β	SE	*p*	R^2
ADHD total traits	5661	0.12	0.10	0.25	-0.06	0.02	0.006	0.0024	0.004	0.033	0.013	0.012	0.001
ADHD hyperactive-impulsive traits	5661	0.16	0.12	0.19	-0.05	0.02	0.031	0.0034	0.002	0.037	0.013	0.005	0.0006
ADHD inattentive traits	5656	0.07	0.13	0.58	-0.04	0.02	0.025	0.037	0.003	0.023	0.013	0.075	0.001
SCDC total score	5653	0.13	0.14	0.36	0.02	0.04	0.66	0.42	<0.001	0.012	0.013	0.36	0.0001
CCC pragmatic language score	5641					N/A				-0.028	0.013	0.038	0.0008

* Linear regression results of ADHD and SCDC phenotypes included only for ease of interpretation.

Polygenic scores derived using a threshold of $p < 0.5$ in the discovery sample GWAS results (see text).

ADHD, attention-deficit/hyperactivity disorder; ALSPAC, Avon Longitudinal Study of Parents and Children; ASD, autism spectrum disorder; CCC, Children's Communication Checklist; SCDC, Social and Communication Disorders Checklist; GWAS, genome-wide association study; ZINB, zero-inflated negative binomial.

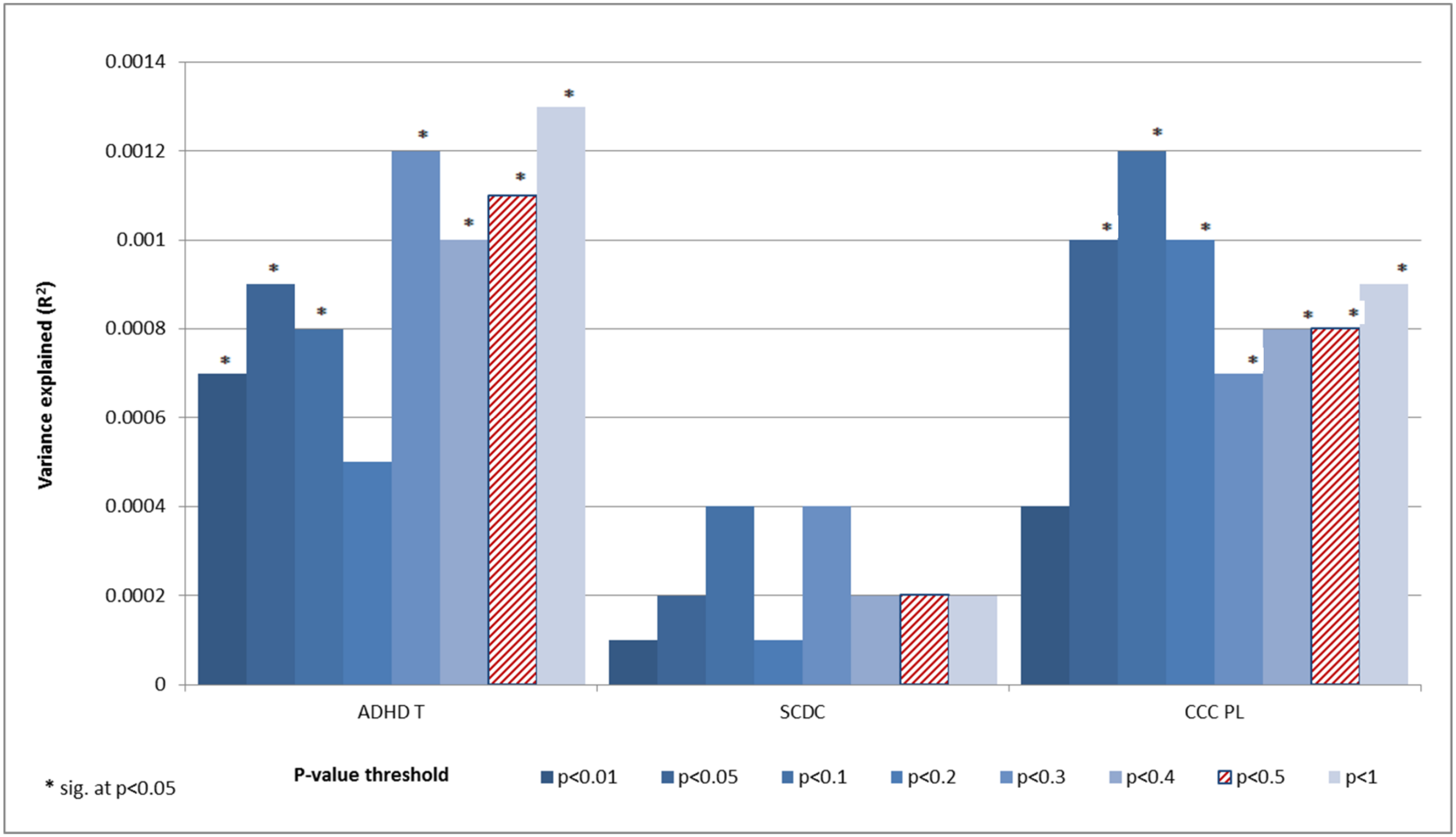

Figure S3. Associations of ADHD and autism spectrum disorder-related phenotypes with ADHD polygenic score calculated based on the primary discovery sample, using a variety of *p*-value thresholds (linear regressions). Main results are based on polygenic scores derived using a threshold of $p < 0.5$ (highlighted). ADHD T: attention-deficit/hyperactivity disorder total traits; CCC PL: Children's Communication Checklist pragmatic language score; SCDC, Social and Communication Disorders Checklist.

Supplemental References

1. Purcell S, Neale B, Todd-Brown K, Thomas L, Ferreira MA, Bender D, *et al.* (2007): PLINK: a tool set for whole-genome association and population-based linkage analyses. *Am J Hum Genet*. 81(3):559-75.
2. Purcell SM, Wray NR, Stone JL, Visscher PM, O'Donovan MC, Sullivan PF, Sklar P (2009): Common polygenic variation contributes to risk of schizophrenia and bipolar disorder. *Nature*. 460(7256):748-52.
3. Ripke S, Sanders AR, Kendler KS, Levinson DF, Sklar P, Holmans PA, *et al*. (2011): Genome-wide association study identifies five new schizophrenia loci. *Nat Genet*. 43(10):969-76.
4. Sklar P, Ripke S, Scott LJ, Andreassen OA, Cichon S, Craddock N, *et al*. (2011): Large-scale genome-wide association analysis of bipolar disorder identifies a new susceptibility locus near ODZ4. *Nat Genet.* 43(10):977-83.
5. Hamshere ML, O'Donovan MC, Jones IR, Jones L, Kirov G, Green EK, *et al.* (2011): Polygenic dissection of the bipolar phenotype. *Br J Psychiatry*. 198(4):284-8.
6. Hamshere ML, Langley K, Martin J, Agha SS, Stergiakouli E, Anney RJ, *et al.* (2013): High loading of polygenic risk for ADHD in children with comorbid aggression. A*m J Psychiatry*. 170(8):909-16.
7. Stergiakouli E, Hamshere M, Holmans P, Langley K, Zaharieva I, Hawi Z, *et al*. (2012): Investigating the contribution of common genetic variants to the risk and pathogenesis of ADHD. *Am J Psychiatry.* 169(2):186-94.
8. Neale BM, Medland SE, Ripke S, Asherson P, Franke B, Lesch KP, *et al*. (2010): Meta-analysis of genome-wide association studies of attention-deficit/hyperactivity disorder. *J Am Acad Child Adolesc Psychiatry.* 49(9):884-97.
